# Rebaudioside D decreases adiposity and hepatic lipid accumulation in a mouse model of obesity

**DOI:** 10.1038/s41598-024-53587-y

**Published:** 2024-02-06

**Authors:** Arianne Morissette, Alice de Wouters d’Oplinter, Diana Majolli Andre, Marilou Lavoie, Bruno Marcotte, Thibault V. Varin, Jocelyn Trottier, Geneviève Pilon, Martin Pelletier, Patrice D. Cani, Olivier Barbier, Vanessa P. Houde, André Marette

**Affiliations:** 1 Cardiology Axis, Québec Heart and Lung Institute (IUCPQ), Université Laval, Québec, QC G1V 0A6 Canada; 2 Institute of Nutrition and Functional Foods (INAF), Université Laval, Québec, Canada; 3 Metabolism and Nutrition Research Group, Louvain Drug Research Institute (LDRI), UCLouvain, Université Catholique de Louvain, Brussels, Belgium; 4 WELBIO-Walloon Excellence in Life Sciences and Biotechnology, WELBIO Department, WEL Research Institute, Avenue Pasteur, 6, 1300 Wavre, Belgium; 5 Institute of Experimental and Clinical Research (IREC), UCLouvain, Université Catholique de Louvain, Brussels, Belgium; 6 Infectious and Immune Diseases Research Axis, Centre de Recherche du CHU de Québec-Université Laval, Québec, Canada; 7 Laboratory of Molecular Pharmacology, Endocrinology and Nephrology Axis, Faculty of Pharmacy, CHU of Québec Research Center, Québec, Canada

**Keywords:** Metabolic syndrome, Obesity

## Abstract

Overconsumption of added sugars has been pointed out as a major culprit in the increasing rates of obesity worldwide, contributing to the rising popularity of non-caloric sweeteners. In order to satisfy the growing demand, industrial efforts have been made to purify the sweet-tasting molecules found in the natural sweetener stevia, which are characterized by a sweet taste free of unpleasant aftertaste. Although the use of artificial sweeteners has raised many concerns regarding metabolic health, the impact of purified stevia components on the latter remains poorly studied. The objective of this project was to evaluate the impact of two purified sweet-tasting components of stevia, rebaudioside A and D (RebA and RebD), on the development of obesity, insulin resistance, hepatic health, bile acid profile, and gut microbiota in a mouse model of diet-induced obesity. Male C57BL/6 J mice were fed an obesogenic high-fat/high-sucrose (HFHS) diet and orally treated with 50 mg/kg of RebA, RebD or vehicle (water) for 12 weeks. An additional group of chow-fed mice treated with the vehicle was included as a healthy reference. At weeks 10 and 12, insulin and oral glucose tolerance tests were performed. Liver lipids content was analyzed. Whole-genome shotgun sequencing was performed to profile the gut microbiota. Bile acids were measured in the feces, plasma, and liver. Liver lipid content and gene expression were analyzed. As compared to the HFHS-vehicle treatment group, mice administered RebD showed a reduced weight gain, as evidenced by decreased visceral adipose tissue weight. Liver triglycerides and cholesterol from RebD-treated mice were lower and lipid peroxidation was decreased. Interestingly, administration of RebD was associated with a significant enrichment of Faecalibaculum rodentium in the gut microbiota and an increased secondary bile acid metabolism. Moreover, RebD decreased the level of lipopolysaccharide-binding protein (LBP). Neither RebA nor RebD treatments were found to impact glucose homeostasis. The daily consumption of two stevia components has no detrimental effects on metabolic health. In contrast, RebD treatment was found to reduce adiposity, alleviate hepatic steatosis and lipid peroxidation, and decrease LBP, a marker of metabolic endotoxemia in a mouse model of diet-induced obesity.

## Introduction

Obesity has reached worrisome proportions worldwide over the last 50 years^1^. Evidence suggests that chronic overconsumption of added sugars, especially in the form of sugar-sweetened beverages, is a major culprit in the obesity pandemic^2^. The consequences of this alarming situation are an ever-growing number of patients with type 2 diabetes (T2D), cardiovascular diseases, non-alcoholic fatty liver disease (NAFLD) and cancer^1^. In an effort to decrease their sugar intake, many consumers have turned towards low- or non-caloric artificial sweeteners (NAS) such as saccharine, aspartame, sucralose or acesulfame potassium (acesulfame K), as they provide a palatable alternative to caloric sugar. Despite the substitution of NAS at the expense of refined sugars, the prevalence of obesity and associated diseases continues to climb^3,4^. Moreover, the long-term impact of such replacement on health is largely unknown and is still a matter of considerable debate^5^. Although some studies have found no or little associations between NAS and body weight^6,7^, others reported that NAS intake is associated with elevated risks of death from all causes^8^ and promotes body weight gain through increasing appetite, hunger, sweets craving, and food intake^3,4^. In addition, a growing number of studies indicate that NAS may disturb the composition of the gut microbiota, a key player in host metabolism^9^. Using gut microbiota transplantation to germ-free mice, Suez et al*.* elegantly demonstrated that the ingestion of saccharin caused glucose intolerance by disrupting gut microbiota^10^. This finding was further validated with healthy adults consuming NAS^11^. In addition, sucralose, aspartame, and acesulfame K have also been reported to impact gut microbiota and metabolism in animal studies (reviewed in Ref.^12^). However, although many studies have raised concerns regarding NAS, the natural non-caloric sweetener stevia appears to be a safer alternative^13^.

*Stevia rebaudiana* is a shrub native to South America, whose leaves contain sweet-tasting molecules called steviol glycosides. The most abundant steviol glycosides are steviosides and rebaudiosides A (RebA), together representing 95% of the total steviol glycosides. Stevia also contains > 40 minor steviol glycosides, notably rebaudioside D (RebD). Once extracted from the plant, steviol glycosides can be used as a non-caloric sweetener, as they are 50–300 times sweeter than sucrose and do not provide any calories^14^. Since RebA is a major steviol glycoside, it was one of the first to be purified and is currently formulated in thousands of food and beverage products^15^. In the past decade, stevia extracts and steviol glycosides have been recognized as safe by the European Food Safety Authority (EFSA) and the U.S. Food and Drug Administration (FDA)^16,17^. In line with this, a systematic review and meta-analysis of randomized clinical trials established that the use of purified RebA does not have any significant effect on blood pressure, fasting blood glucose or lipid profile, hence supporting the idea that steviol glycosides represent a safer alternative among the NAS^18^.

In contrast to synthetic molecules, steviol glycosides have been very well received by consumers^19,20^. Nevertheless, many consumers remain reluctant towards stevia because of its taste. RebA, which is the most commonly used purified steviol glycoside, has a lingering bitter, licorice flavour, which gets worse at higher concentrations^15,20^. On the other hand, the minor steviol glycoside RebD is characterized by a cleaner, sweeter taste and decreased bitterness^21^. However, since it only represents a minor proportion of steviol glycosides, only a negligible amount of RebD is naturally present in stevia. Manufacturers have recently identified strategies to efficiently biosynthesize RebD for its incorporation in food and beverages^22^. Yet, the impact of purified RebD at quantities comparable to those of purified RebA on metabolic health and gut microbiota has never been investigated. The latter is of major importance, as all steviol glycosides require the action of gut microbiota to be degraded and further eliminated by the host through urine or feces^23^. In addition, is it now recognized that the gut microbiota plays a key role in maintaining metabolic homeostasis^24^.

In an effort to identify safe and efficacious ways to limit the consumption of added sugars, we aimed to investigate the impact of RebA and RebD on metabolic health in a mouse model of obesity. We also aimed to thoroughly investigate the impact of these rebaudiosides on gut microbiota using whole shotgun metagenomic sequencing.

## Methods

### Animals

Animal experiments reported in this manuscript comply with the Animal Research: Reporting of In Vitro Experiments (ARRIVE) guidelines. Eight-week-old C57Bl/6 J male mice (Jackson, Bar Harbor, ME, USA) were individually housed in a controlled environment (12 h day/night cycle, lights off at 18:00) with food and water ad libitum in the animal facility of the Quebec Heart and Lung Institute (Québec, Qc, Canada). Mice were randomly assigned to a chow diet or a high-fat high-sucrose (HFHS) diet for 12 weeks. The protein sources in the HFHS diet (23% beef, 24% chicken, 23% pork, 11% soy, 6% egg and 11% casein) were designed to match the protein consumption in a typical human diet based on the National Health and Nutrition Examination Survey 2011–2012^25^ (diet composition in Table 1). The different protein sources were purchased from various vendors, as detailed in^26^. The first group of mice fed chow was daily gavaged with vehicle (water) (n = 19). A second group of mice fed HFHS were also daily gavaged with vehicle (n = 20). Two other groups were also fed HFHS but daily gavaged with either 50 mg/kg/day of RebA (n = 20) or RebD (n = 17). The quantity daily given was based on the acceptable daily intake for humans (4 mg/kg/day) adjusted for mice, which represents approximately 10 packets of tabletop stevia sweetener per day for a 150 lbs (68 kg) individual. Fresh feces were collected at weeks 0 and 12 and kept at -80 degrees Celsius until bacterial genomic DNA was extracted. The food intake was measured 3 times a week and body weight was assessed twice a week. At week 12, animals were anesthetized in chambers saturated with isoflurane and then euthanized by cardiac puncture. Tissues were harvested and blood was drawn in tubes coated with heparin and centrifuged. This study followed the guide for the care and use of laboratory animals and all procedures had been previously approved by the Laval University Animal Ethics Committee (CPAUL #2018023-1).

**Table 1:** High-fat high sucrose diet composition.

Ingredients	g/100 g	Energy (%)	Total energy per macronutrient (%)
Protein mix	22.3	14.8	15
L-cystine	0.3	0.2	
Starch	6.5	4.9	35
Sucrose	34.07	28.3	
Mineral mix	6.7	0.7	
Vitamin mix	1.4	1.1	
Cellulose	5		
Lard	15	28.0	50
Corn oil	8.4	15.7	
Fat from protein mix		6.3	
Choline bitartrate	0.3		
BHT	0.03		
Total	100	100	100
Total calories	4.82 kcal/g		

### Glucose homeostasis

At 10 weeks, mice were fasted for 6 h and were subjected to an intra-peritoneal insulin tolerance test (ITT) (0,65U/kg body weight). Blood was collected by the caudal vein and glycemia was measured with an Accu-Check glucometer (Bayer) before (0 min) and after (5, 10, 15, 20, 30, 60 min) intraperitoneal insulin injection. At the end of week 12, mice were fasted overnight (12 h) and subjected to an oral glucose tolerance test (OGTT) (1 g of glucose/kg body weight). Blood was collected by the caudal vein before (0 min) and after (15, 30, 60, 90, 120 min) glucose bolus for measuring glycemia and insulinemia. Mice were gavaged with RebA, RebD or vehicle 2 h before the tests.

### Analytical methods

Plasma insulin concentration was measured using an ultra-sensitive ELISA kit (Alpo, Salem, NH, USA and Chrystal Chem, Elk Grove Village, IL, USA, respectively). Liver triglycerides and cholesterol were extracted with chloroform–methanol. Hepatic triglyceride and cholesterol content were measured by enzymatic reactions with commercial kits (Randox Laboratories). Circulating levels of triglycerides, alanine aminotransferase, and aspartate aminotransferase were quantified using the Flex TRIG, ALTI, and AST reagent cartridge, respectively, on the Dimension Vista 1500 system (Siemens, Germany). Fecal short-chain fatty acids (SCFA) and branched-chain fatty acids (BCFA) were assessed by gas chromatography as previously described^27^. Fecal pellets collected at week 10 were dried and gross energy density was determined using adiabatic bomb calorimetry (Parr Instruments, Moline, IL, USA). Lipid peroxidation was assessed by Thiobarbituric acid reactive substances (TBARS) assay kit (R&D Systems). Lipopolysaccharide binding protein (LBP) was measured by ELISA (Hycult biotech).

### Quantitative reverse transcriptase PCR (qPCR) of liver tissue

Livers were snap-frozen and reduced to powder using a mortar, pestle, and liquid nitrogen. The ileum was snap-frozen, and RNA was extracted from these tissues using the Directzol RNA miniprep kit (Zymo Research). cDNA synthesis was made from 1ug of RNA using a High-capacity cDNA Reserve Transcriptase kit (Applied Bioscience) following the manufacturer’s instructions. qPCR was carried out using 4 μl of cDNA, 5 μl of Advanced qPCR MasterMix (Wisent Bioproducts) and 0.5 μl of each primer (diluted at a concentration of 10 μM) in a total reaction volume of 10 μL with the following cycle setting: 95 °C for 2 min (95 °C for 20 s, 61.5–62 °C for 20 s, 72 °C for 20 s) × 40 ending with a melting Curve: 65 °C to 95 °C. Each target gene was evaluated and accepted in the case of a unified peak from melting curves and efficiency of 100% ± 15. Relative expression was calculated by 2^ΔCq^ of target Cq to reference gene Cq of the sample accepting replicates with a coefficient of variation < 0.03. Reference genes used were Actin (*actb*), Glyceraldehyde 3-phosphate dehydrogenase (*gapdh*) and Hypoxanthine Phosphoribosyltransferase 1 (*hprt*) for the liver, and Actin and Gapdh for ileum. The list of primers is provided in Supplementary Table 1. Statistical analyses were performed on the expression of genes.

### Immunoblotting

Liver and gastrocnemius muscle were powdered with liquid nitrogen and then homogenized under rotation for 2-h at 4 °C in a tenfold mass excess of ice-cold lysis buffer (50 mM Hepes pH 7.5, 150 mM NaCl, 1 mM EGTA, 20 mM β-glycerophosphate, 1% NP-40, 10 mM NaF, 2 mM Na3VO4, 0.1 mM PMSF and protease inhibitors cocktail). Lysates were clarified by centrifugation at 16,000 × *g* for 10 min at 4 °C and the proteins were measured with a BCA assay (ThermoFisher Scientific, Burlington, Canada). Tissue lysates (5–30 μg) were denatured in SDS sample buffer and submitted to SDS-PAGE followed by transfer on nitrocellulose membranes (Pall Corporation, Mississauga, Canada). Membranes were blocked for 1-h at room temperature and then probed with the primary antibody overnight at 4 °C. After washing in TBST (50 mM Tris–HCl pH 7.5, 0.15 mM NaCl, and 0.1% Tween-20), the membranes were incubated with HRP-conjugated secondary antibodies for 1-h at room temperature. Immunoblots were analyzed using ImageJ software (NIH, Bethesda, USA). The primary antibodies used were Anti-actine, clone C4, EMD Millipore Corp., MAB1501; Acetyl-CoA carboxylase, Cell Signaling Technology, 3662S; and phospho(Ser79)-Acetyl-CoA carboxylase, Cell Signaling Technology, 3661S.

### Bile acid quantification

Bile acids from feces, plasma and liver were quantified by LC–MS/MS as previously described, with slight modifications^28^. Briefly, lyophilized fecal samples (2 mg) were homogenized in 500μL of water: methanol (50:50) containing 0.1% formic acid solution. Fifty microliters of internal standard (mix of CDCA-d4, DCA-d4, CA-d4, LCA-d4, TCA-d5 and GCA-d4; C/D/N Isotopes Montréal, Canada) were added. Homogenates were centrifuged at 5000 *g* for 5 min. Supernatants were then evaporated under nitrogen and resuspended in 2 mL of a water-0.1% formic acid solution, before performing solid phase extraction (SPE) using a pre-conditioned (methanol and water-0.1% formic acid) Strata-X 60 mg columns (Phenomenex, Torrance, CA, USA). SPE columns were washed with water (2 mL) and a water: methanol solution (80:20) containing 0.1% formic acid (2 mL). Analytes were then eluted using 2 mL methanol. Elutes were evaporated under nitrogen and reconstituted in 100 μL water: methanol (50:50) prior to injection. One μL of samples or analytical standards was then injected into the chromatographic system consisting of a Prominence high-pressure liquid chromatography (HPLC) instrument (Shimadzu Scientific Instruments, Columbia, MD, USA). The chromatographic separation was achieved with a C18 column from Agilent (150 × 2.1 mm Poroshell 120 EC-C18; 2.7 μm particles; Santa Clara, CA) at 45 °C, and the following mobile phases: solvent A = ammonium acetate in water (6 mM) at pH 7.7 and solvent B = acetonitrile. Separation was performed at a flow rate of 0.35 mL/min using the following sequence: 84% A:16% B as initial conditions held for 0.5 min and increased to 27% B in 16.5 min, then a linear gradient to 31% B over the next 16 min, followed by an increase of B to 56% in 8 min. The column was then flushed at 95%B over the next 12 min and back to initial conditions for 8 min. All analytes were quantified by tandem mass spectrometry (MS/MS) using an API4000 instrument (Applied Biosystems, Concord, ON, Canada). The temperature was set at 500 °C. The same procedure was used for liver samples (20 mg) except using 500 μL of methanol containing 0.1% formic acid as the homogenizing solution. Plasma samples (50 uL) were diluted in 2 mL of a water-0.1% formic acid solution containing 50 µl of the internal standard mix before performing SPE and HPLC–MS/MS analysis as described above.

### Caecal short-chain fatty acid quantification

Phosphoric acid was purchased from VWR. Diethyl ether (99.5%) and all the 99% grade standards (acetic acid, propionic acid, isobutyric acid, butyric acid, isovaleric acid, valeric acid and internal standard 4-methyl valeric acid) were purchased from Sigma-Aldrich. Cecum SCFA was quantified by gas chromatography. Caeca were collected and kept frozen at − 80 °C until extraction. After the addition of 1 mL H_2_O per 100 mg of material, the suspensions were homogenized for 2 min and then centrifuged at 18,000 *g* for 10 min at 4 °C. The supernatants were spiked with 4-methylvaleric acid and acidified with phosphoric acid 10%. To extract SCFAs, samples were mixed for 2 min with an equal volume of diethyl ether and then centrifuged at 18,000 *g* for 10 min at 4 °C. Organic phase analysis was performed on a GC-FID system (Shimadzu), constituted of a GC 2010 Plus gas chromatograph equipped with an AOC-20 s auto-sampler, an AOC-20i auto-injector and a flame ionization detector. The system was controlled by GC solution software. SCFA were separated on a Nukol capillary GC column (30 m × 0.25 mm id, 0.25 µM film thickness, Supelco analytical). The column flow was constant at 1.3 mL/min of hydrogen. The injector was set at 230 °C and the detector at 250 °C. The oven temperature was initially programmed at 60 °C, then increased to 200 °C at 12 °C/min, and held for 2 min. SCFA was quantified using a 5-point calibration curve prepared with a mix of acetic acid, propionic acid, butyric acid, isobutyric acid, valeric acid, isovaleric acid, and internal standard 4-methyl valeric acid.

### Oil Red-O staining

During the sacrifice, mouse livers were embedded in Tissue-Tek OCT, immediately snap-frozen in liquid nitrogen, and stored at − 80 °C. The staining of neutral lipids was based on the methods described by Mehlem et al.^29^. Briefly, 10 mm liver sections were allowed to equilibrate at room temperature for 5 min and then postfixed with a Formalin (10%)/Calcium (2%) solution for 15 min. The sections were then incubated with Oil-Red O (ORO) working solution at room temperature for 5 min, followed by 5 min of clearing in 60% isopropyl alcohol and a counterstaining of 15 s with Mayer’s hematoxylin. Pictures of liver sections were taken with a wide-field microscope (Zeiss) within 30 min after staining with a 20 × objective. Quantification of ORO-positive area was done with Image-Pro Plus software. To calculate the mean ORO-positive area, eight pictures of each liver section were quantified.

### Microbial genomic DNA extraction and whole-genome shotgun sequencing

Bacteria genomic DNA was extracted from fresh fecal samples collected at week 12 with the ZymoBIOMICS DNA miniprep kit (Zymo Research) according to the manufacturer’s instructions. Metagenomic libraries were prepared with NEBNext Ultra II DNA Library Prep Kit for Illumina (New England BioLabs) as per the manufacturer’s recommendations. Libraries were quantified using the Kapa Illumina GA with Revised Primers-SYBR Fast Universal kit (Kapa Biosystems). The average size fragment was determined using a LabChip GX (PerkinElmer) instrument. Libraries were sequenced on the Illumina NovaSeq 6000 platform using a 2 × 150 bp paired end run. Metagenomic reads were quality-filtered using Trimmomatic with a cutoff ≥ Q20^30^ and further filtered to remove the host-origin reads using Bowtie2^31^. Host-decontaminated reads were taxonomically profiled at the species level using Kraken2^32^ and the abundance of species in annotated reads from a metagenomic sample was computed using Bracken^33^. Species that were not present in at least 10% of all samples were discarded. Functional analyses were performed using HUMAnN3^34^, and KEGG pathways/functions were generated from HUMAnN3 output.

The R Phyloseq package (v1.26.1; Ref.^35^) was used to perform all diversity analyses. Shannon and Simpson diversity indices were used to reflect the richness and evenness of microbial representation in a sample. Additionally, the similarity of microbial communities between samples was calculated using principal component analysis (PCA) based on the Aitchison distance. Detection of differentially abundant taxa or pathways between groups was performed with the metagenomics biomarker discovery tool LefSe (Linear Discriminant Analysis Effect Size) using an LDA score threshold > 2^36^.

### Statistical analysis

Data are expressed as mean ± SEM. One-way ANOVA with the Dunnett post-hoc test was used to assign significance to the comparison between RebA or RebD to the HFHS control group. When the homoscedasticity was significantly different between groups according to Bartlett’s test, a Welch ANOVA was performed and Dunnett’s T3 test was used to assign significance. When normality was not respected, ANOVA on ranks with Dunn’s post-hoc test was used. Time points within different groups were compared using two-way repeated measures ANOVA with Tukey’s post-hoc test. When missing values were detected, mixed model analysis was performed (Sigma Plot, San Jose, CA, USA). Multiple comparison results were considered statistically significant at p < 0.05.

## Results

### RebD decreases adipose depots, and steviol glycosides do not deteriorate glucose homeostasis

**Figure 1:**
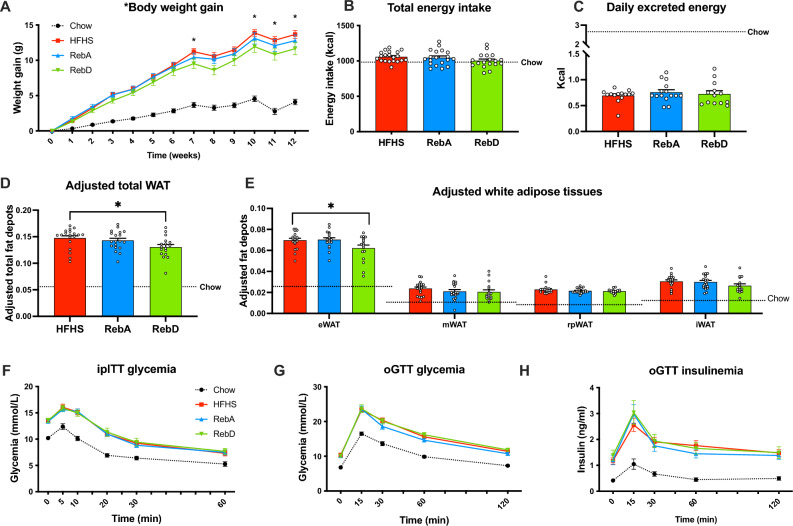
Daily gavage with RebD decreases weight gain and adiposity, and RebA and RebD do not alter glucose homeostasis. (**A**) Body weight gain, (**B**) total energy intake, and (**C**) daily fecal energy excretion. (**D**) Total adjusted adipose tissue, namely (**E**) epidydimal (eWAT), mesenteric (mWAT), retroperitoneal (rpWAT), and inguinal fat (iWAT) adjusted for body weight. (**F**) Glycemia during intraperitoneal insulin tolerance test (ipITT). (**G**) Glycemia and (**H**) insulinemia during oral glucose tolerance test (OGTT). Data are expressed in mean ± SEM. n = 17–20. (**B**–**D**) One-way ANOVA test with Dunnett’s post-hoc. (**E**) One-way Welch’s ANOVA with Dunnett’s T3 multiple comparisons test. (**A**,**F**–**H**) Two-way ANOVA with Tukey’s post-hoc. *p < 0.05 HFHS vs. RebD.

For 12 weeks, mice were fed chow or HFHS and daily gavaged with either vehicle (water), or 50 mg/kg of RebA or RebD. As expected, mice fed HFHS gained substantially more weight than mice fed chow. RebD-administered mice, but not RebA-administered mice, gained significantly less weight than the HFHS control at weeks 8 and 10 through 12 (Fig. 1A). The total caloric intake and fecal energy excretion were unchanged between all HFHS-fed mice and therefore could not explain why mice receiving RebD had a decreased body weight gain (Figs. 1B,C). Mice were then sacrificed, and adipose tissues were collected and weighed. The sum of all white adipose tissues was significantly lower in mice receiving RebD as compared to HFHS-fed mice (Fig. 1D). This finding was evidenced by a specific decrease in epididymal adipose tissue (Fig. 1E). No change in BAT weight was detected (data not shown).

Since it was previously reported that some artificial sweeteners can alter glucose homeostasis, we investigated whether RebA and RebD could also perturb insulin sensitivity and glucose tolerance^10^. At week 10, an intraperitoneal insulin tolerance test was performed. As depicted in Fig. 1F, no differences in insulin tolerance were observed between HFHS-fed mice, therefore suggesting that RebA and RebD do not alter insulin sensitivity. At week 12, mice were subjected to an oral glucose tolerance test. HFHS-fed mice daily gavaged with RebA or RebD showed similar glucose intolerance compared to mice gavaged with the vehicle (Fig. 1G). Moreover, no significant changes were observed for glucose-induced insulin responses (Fig. 1H). These results therefore indicate that daily gavage with steviol glycosides does not exacerbate HFHS-induced glucose intolerance and insulin resistance.

### RebD decreases triglyceride and cholesterol accumulation in the liver, and limits lipid peroxidation

**Figure 2:**
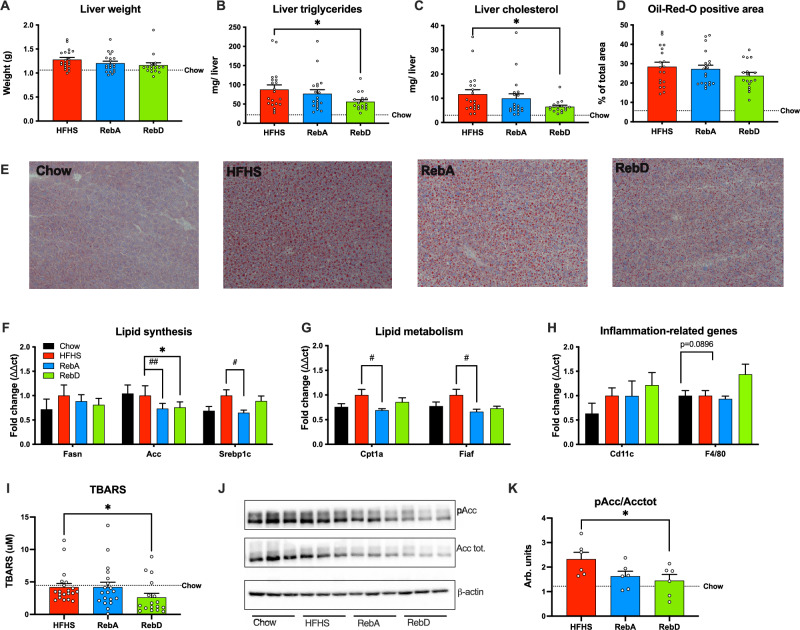
RebD attenuates the accumulation of hepatic triglycerides and cholesterol. (**A**) Liver weight. (**B**,**C**) Liver triglyceride and cholesterol accumulation. (**D**) Quantification of Oil-red O (ORO)-positive area. Representative images of hepatic lipid accumulation by ORO staining at 20X objective for (**E**) chow, HFHS, RebA and RebD. Liver mRNA expression of genes related to (**F**) lipid synthesis, (**G**) lipid metabolism, and (**H**) inflammation were quantified by RT-qPCR; HFHS group as the control. (**I**) Thiobarbituric acid substances (TBARS) are measured in the liver. (**J**) Cropped immunoblotting and (**K**) quantification of densitometry analysis for phosphorylated Acetyl-CoA Carboxylase (pSer79-Acc) corrected for total Acc. Original Blots/gels are presented in Supplementary Fig. 2 and 3. (**F**) One-way ANOVA with Dunnett’s post-hoc. (**B**,**C**,**G**,**K**) One-way Welch’s ANOVA with Dunnett’s T3 multiple comparisons test. (**I**) Kruskal–Wallis test with Dunn’s multiple comparisons test. Data are expressed as the mean ± SEM. n = 17–20. *p < 0.05 HFHS vs. RebD. ^#^p < 0.05 HFHS vs. RebA. ^##^p < 0.01 HFHS vs. RebA.

The liver was collected at sacrifice, and no significant differences in organ weight were detected between all HFHS-fed mice (Fig. 2A). Lipid extraction from the liver revealed that mice receiving RebD accumulated less triglycerides and cholesterol (Fig. 2B,C). To further investigate this finding, Oil-Red O (ORO) staining was performed and showed that mice administered steviol glycosides, especially RebD, had attenuated hepatic lipid accumulation by roughly 5% (Fig. 2E), although the quantification did not reach significance (Fig. 2D). Plasma levels of triglycerides, cholesterol, ALT and AST were measured, and no significant differences between groups were detected (Supplementary Fig. 1).

Consistent with the improved hepatic steatosis, the mRNA expression of genes related to lipid synthesis was decreased by steviol glycosides, where the expression of acetyl-CoA Carboxylase (*accA*) was decreased by both RebA and RebD and the sterol regulatory element binding protein 1 (*srebp1c*) by RebA only (Fig. 2F). In addition, phosphorylation of Acetyl-CoA Carboxylase (pAcc) corrected for total Acc protein abundance (Acc tot) was decreased in the livers of RebD mice (Fig. 2J–K). Uncropped images can be found in the Supplementary Material (Supplementary Figs. 2 and 3). The mRNA expression of carnitine palmitoyltransferase 1A (*cpt1a*), involved in the mitochondrial oxidation of lipids, and fasting-induced adipose factor (*Fiaf*), an inhibitor of the lipoprotein lipase, were decreased by RebA (Fig. 2G). No changes in the expression levels of the pro-inflammatory genes *cd11c* and *F4/80* were observed, although a trend was observed for *F4/80* (P = 0.0896) (Fig. 2H). Finally, the levels of TBARS, a marker of lipid peroxidation, were decreased in the livers of RebD mice (Fig. 2I), which supports the hepatoprotective action of RebD.

We have performed RT qPCR analysis to investigate the impact of RebD on colonic markers of gut permeability but have not observed any significant changes (Supplementary Fig. 4). However, as colons were collected at sacrifice and mice were fasted overnight, the impact of RebD on the gene expression may be transient and prevailed us from observing any significant changes.

### RebA and RebD modify bile acid metabolism

**Figure 3:**
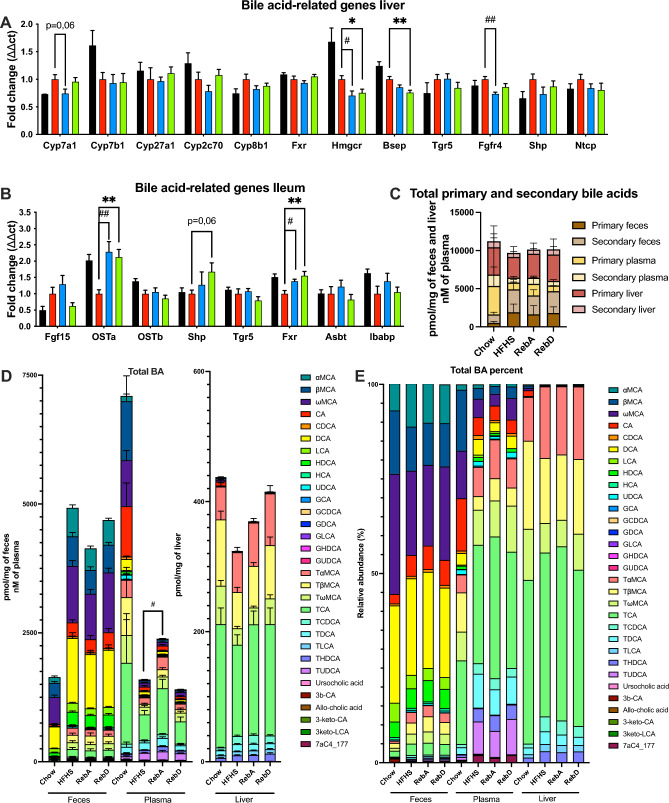
RebA and RebD modify bile acid metabolism. Messenger RNA extracted from the liver (**A**) and ileum (**B**) of genes involved in bile acid metabolism was quantified by RT-qPCR. (**C**) Total primary and secondary bile acids in feces, plasma, and liver. (**D**,**E**) Bile acids measured in feces, plasma, and liver are expressed in absolute and relative abundance. (**A**,**B**,**D**) One-way ANOVA with Dunnett’s post-hoc test. Data are expressed as the mean ± SEM. n = 17–20. *p < 0.05 HFHS vs. RebD. **p < 0.01 HFHS vs. RebD. ^#^p < 0.05 HFHS vs. RebA. ^##^p < 0.01 HFHS vs. RebA.

Since BA has been shown to regulate energy homeostasis and lipid metabolism^37^, we next evaluated whether changes in BA-related genes and BA pool could be linked to the metabolic effects seen in mice administered RebA and RebD. In the liver, both rebaudiosides decreased the gene expression of HMG-CoA reductase (*hmgcr*), the rate-controlling enzyme involved in cholesterol synthesis, which is the main constituent of BAs (Fig. 3A). In the ileum, RebA and RebD increased the mRNA expression of the Farnesoid X receptor (*fxr*). FXR is a master regulator of BA homeostasis and controls BA levels by acting on several target genes in the enterohepatic circulation. More precisely, hepatic BA synthesis is controlled by a feedback mechanism orchestrated by the FXR-mediated induction of fibroblast growth factor 15 (FGF15) in the small intestine. FGF15 then binds to the fibroblast growth factor receptor 4 (FGFR4), which contributes to the downregulation of BA synthesis. Intestinal FXR also regulates the expression of bile acid transporters, thereby controlling BA formation and recycling. In line with this, RebA and RebD increased the mRNA expression of the organic solute transporter (*ost*)α in the ileum, suggesting an efflux of BA in the portal vein (Fig. 3B). When reaching the liver, BA and FGF15 suppress BA synthesis through inhibition of the rate-limiting enzyme CYP7A1 involved in BA production. RebA nearly significantly decreased the expression of *cyp7a1*, and RebA and RebD decreased the expression of the bile salt export protein (*bsep*), suggesting a decreased production and export of BA from the liver (Fig. 3A).

Bile acids were then measured in the feces, plasma, and liver of mice. No changes in the total levels of primary and secondary bile acids were observed in the three tissues among HFHS-fed mice (Fig. 3C). However, mice administered RebA had increased plasma levels of total bile acids (Fig. 3D). This could be explained by the decreased expression of FGFR4, which contributes to the downregulation of BA synthesis (Fig. 3A). Moreover, RebA increased the plasma levels of tauro-β muricholic acid (TβMCA) and taurohydroxydeoxycholic acid (THDCA) compared to HFHS-fed mice, which are respectively primary and secondary BAs and decreased the fecal level of the secondary BA LCA (Supplementary Fig. 5A,B). RebD tended to increase the hepatic level of the primary BA TβMCA, which can be converted into the secondary BAs TωMCA and ωMCA, which both tended to be increased in the liver (Supplementary Fig. 5C). No changes in the relative abundance of BA between groups were observed (Fig. 3E).

### RebD induces changes in the gut microbiota taxonomy and pathways and decreases LBP, a marker of metabolic endotoxemia

**Figure 4:**
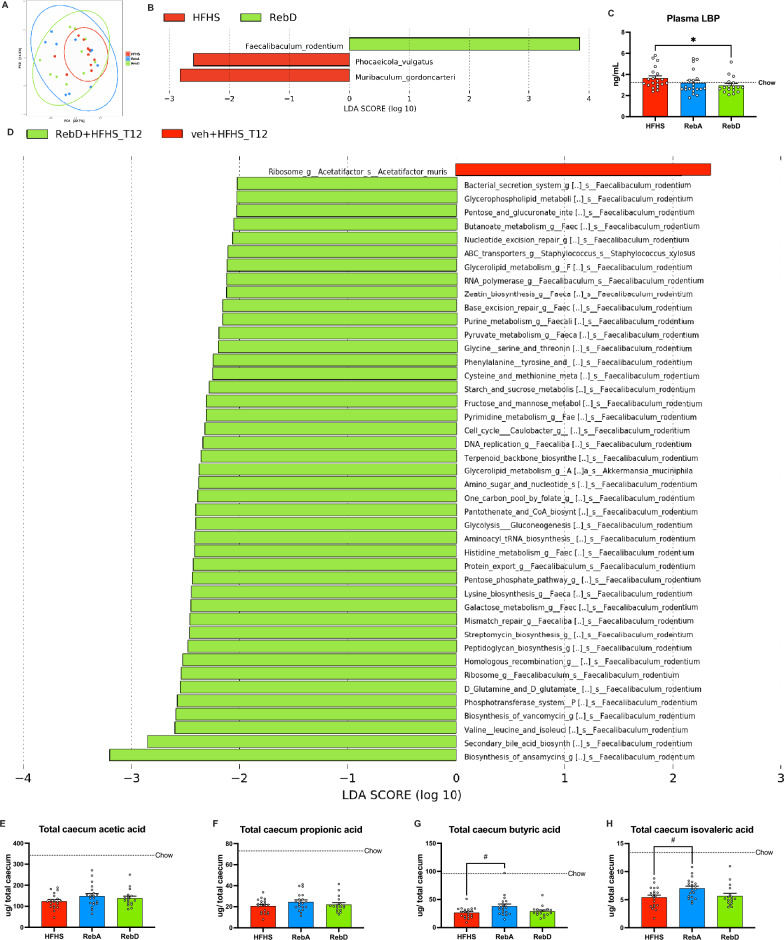
RebD, but not RebA, induces changes in the taxonomy and pathways of the fecal gut microbiota and decreases LBP. Genomic DNA was extracted from fecal samples and analyzed by whole-genome shotgun sequencing. (**A**) PCA of the Aitchison distance between groups. (**B**) Linear discriminant analysis (LDA) effect size (LEfSE) was calculated to identify differentially abundant taxa between the gut microbiota of HFHS-fed mice administered the vehicle and RebD. (**C**) Plasmatic levels of lipopolysaccharide-binding protein (LBP). (**D**) Bacterial pathways differentially expressed by bacteria of HFHS-fed mice administered the vehicle or RebD calculated by LEfSE. Ceacum concentration of (**E**) acetic, (**F**) propionic, (**G**) butyric, and (**H**) isovaleric acid. n = 17–20 for SCFA measurements. n = 10 for shotgun sequencing. Data are expressed in mean ± SEM. (**D**) Kruskal–Wallis test with Dunn’s post-hoc. (**E**–**H**) One-way ANOVA with Dunnett’s post-hoc. *p < 0.05 HFHS vs RebD. ^#^p < 0.05 HFHS vs RebA.

We next assessed the impact of administering rebaudiosides on gut microbiota by using whole-genome shotgun sequencing (Supplementary Fig. 6). Fecal samples were collected following the 12-week intervention. A principal component analysis of taxonomy revealed that HFHS-fed mice gavaged either with the vehicle, RebA or RebD clustered close to one another (Fig. 4A). When analyzing the fecal microbiota by Linear Discriminant Analysis (LDA) effect size (LEfSe), it was revealed that the fecal microbiota of mice administered RebD harboured significantly more *Feacalibaculum rodentium*, whereas the feces of mice administered the vehicle contained a higher abundance of *Muribaculum gordoncarteri* and *Phocaeicola vulgatus* (Fig. 4B). No significant differences were detected between the composition of the gut microbiota of mice gavaged with RebA and the vehicle. The circulating levels of lipopolysaccharide-binding protein (LBP), a marker of gut permeability and metabolic endotoxemia, were significantly decreased by RebD (Fig. 4C).

In addition to changes in the taxonomy, whole-genome shotgun sequencing of fecal microbiota revealed that mice administered RebD presented with several enriched bacterial pathways. Indeed, community functional profiles stratified by identified bacteria revealed that *Faecalibaculum rodentium* was responsible for the upregulation of several pathways, including those related to the production of antimicrobial compounds and the metabolism of secondary bile acids. No bacterial metabolic pathways were upregulated by the administration of RebA compared to mice administered with the vehicle (Fig. 4D).

Since *Faecalibaculum rodentium* is known to be an important butyrate producer, we next investigated whether the administration of rebaudiosides altered the concentrations of caecal short-chain fatty acids (SCFA) and branched-chain fatty acids (BCFA). Daily administration of RebD did not alter the caecal concentration of SCFA and BCFA (Fig. 4E–H). However, RebA significantly increased the caecal concentration of butyric acid and isovaleric acid (Fig. 4E,G).

## Discussion

This study shows that the daily administration of 50 mg/kg of RebD attenuated body weight gain and visceral adiposity, decreased liver steatosis and circulating levels of LBP, a marker of metabolic endotoxemia, and increased the abundance of *Faecalibaculum rodentium*. The whole genome shotgun sequencing also revealed that RebD increased the bacterial metabolic pathway related to secondary bile acid metabolism. In line with this, several genes in the liver and ileum involved in bile acid metabolism were modulated following the administration of RebD and RebA.

Although they were also fed an obesogenic diet, daily administration of RebD decreased body weight gain, leading to a statistically significant reduction of epididymal fat mass. RebA treatment did not alter weight gain or fat depots, which is in line with recent findings suggesting that daily consumption of 2–3 mg/kg of RebA in drinking water did not alter body weight gain, nor adiposity in rats fed low-fat or high-fat diets^38,39^. These results thus demonstrate that daily administration of RebA or RebD does not accentuate diet-induced weight gain as previously observed with other types of NAS^9^. Moreover, the fact that RebD decreases HFHS-induced adiposity suggests that it might be a better alternative than RebA for its use as a natural sweetener in addition to providing an improved taste^21,40^.

It was previously demonstrated that different NAS can induce glucose intolerance in both animals and humans^10,11^. Hence, it is important to assess to what extent steviol glycosides can influence glucose homeostasis. While the subject remains debated, most studies point towards a neutral or beneficial effect of stevia or its purified steviol glycosides on metabolic health. Indeed, previous animal studies have proposed that steviosides, which is another type of purified steviol glycoside, could reduce blood glucose levels by stimulating insulin secretion and decreasing blood glucagon levels in animal models^41–43^. On the other hand, other long-term studies have found that administration of RebA does not alter glucose tolerance in rats^38,39^. In humans, a systematic review and meta-analysis suggested that the administration of steviol glycosides slightly decreased fasting blood glucose, although this finding did not reach statistical significance, and no changes were observed for glycated hemoglobin (HbA1c)^13^. The potential metabolic effects of RebD have, however, never been reported. In the present study, in HFHS-fed mice, RebA and RebD did not deteriorate insulin and glucose tolerance. Hence, our data indicate that both RebA and RebD are safe sweetener alternatives regarding glucose homeostasis, at least in diet-induced obese mice.

The gut microbiota plays a key role in metabolic health^44^. An increasing body of literature indicates that artificial non-nutritive sweeteners are associated with metabolic disorders, which stem from alterations to the gut microbiota composition, function and metabolome^9^. As the metabolism of steviol glycosides is dependent upon gut microbiota, it was crucial to determine the effect of RebA and RebD on the bacterial communities residing in the intestines. Indeed, steviol glycosides are undigested in the upper gastrointestinal tract and are hydrolyzed and degraded only when in contact with gut microbiota. Microbes such as Bacteroides species are known to cleave the glycosidic linkages, removing sugar moieties, and leaving behind the steviol backbone that is systematically absorbed, glucuronidated in the liver, and excreted in the feces in rodents^19^. As the sugar moieties are not absorbed, they are most likely utilized by the gut microbes as an energy source. Although this has never been demonstrated, these sugar moieties could be used by F. rodentium as an energy substrate, thus increasing its abundance.

When analyzing the composition of the gut microbiota by whole-genome shotgun sequencing, it was revealed that HFHS-fed mice administered RebD harboured significantly more *Faecalibaculum rodentium* than the HFHS control group. In line with other animal studies, F. rodentium is associated with improved metabolic outcomes^45–52^. It was reported that F. *rodentium* possesses several genes related to the production of SCFA^53,54^. Numerous animal studies and a handful of human studies suggested a beneficial role of these metabolites in the prevention and treatment of obesity and its comorbidities by acting as signaling molecules on energy expenditure, lipid oxidation and leptin secretion^55^. Supplementation of acetate, propionate or butyrate to animals reduced hepatic fat accumulation, decreased hepatic inflammation, and suppressed cholesterol synthesis, and the underlying mechanisms are related to increased hepatic lipid oxidation via an AMPK–acetyl-CoA carboxylase pathway, reduced tumour necrosis factor (TNF) expression, increased glycogen storage and reduced hepatic fatty acid synthase activity.^56–61^. Although we did not observe significant changes in the concentration of SCFAs in the caecum of mice administered RebD, given that SCFAs can be absorbed in the systematic circulation, whether caecal concentration is the best predictor of microbial SCFA production is debatable. A study has even suggested that in rodent experiments with isotopically labelled SCFAs, the absorption of SCFA, not the caecal concentration of SCFA, is relevant for metabolic health^62^.

The strictly anaerobic bacterium *F. rodentium* uses mucus as an ecological niche and was negatively associated with intestinal tumour growth^63^. Hence, the increased abundance of *F. rodentium* could favour a thicker mucus layer and, thus, improve the gut barrier and gut health. Additionally, we previously reported that the administration of prebiotics to HFHS-fed mice was associated with a bloom of F. rodentium and regulated key mucosal markers involved in the repair of epithelial barrier integrity, thereby attenuating circulating levels of LBP, obesity-associated gut dysbiosis and metabolic inflammation^50^. We, therefore, believe that the administration of RebD could have protected the gut barrier function and improved immunomodulation by exerting modulatory effects on the gut microbiota composition. This is evidenced by decreased levels of circulating LBP and TBARS, a marker of lipid peroxidation. The taxonomic analysis also revealed that the microbiota of the HFHS-fed control group harboured significantly more *Phocaeicola vulgatus*, also known as *Bacteroides vulgatus*^64^. This bacterium, along with *Prevotella copri*, has been identified as the main species driving the association between the biosynthesis of microbial branched-chain amino acids and insulin resistance^65^.

Metagenomic shotgun sequencing also revealed that several bacterial metabolic pathways were upregulated following RebD administration, such as the secondary bile acid metabolism by *F. rodentium*. BAs are amphipathic steroid metabolites facilitating the intestinal absorption of lipids and fat-soluble vitamins. They are also important signaling molecules virtually reaching every organ in the body to fine-tune metabolic functions^66^. The production and diversity of BAs depend on the host and microbial metabolism^67^. Primary bile acids are synthesized in the liver and can be conjugated to taurine (in mice) or glycine (in humans). They are then secreted in the bile and released into the intestinal lumen after food ingestion. In the gut, primary BAs are metabolized into secondary BAs under the action of the gut microbiota through different reactions, including deconjugation, 7α-dehydroxylation, 6α-hydroxylation and epimerization. The vast majority of BAs are reabsorbed by the gut epithelium and return to the liver through the enterohepatic circulation, while a minor fraction transits through the colon and is excreted in the feces^68^. Our data show that mice administered RebD tended to have higher levels of the secondary BAs TωMCA and ωMCA in their livers. In line with our findings, administration of a symbiotic mixture increased the abundance of *F. rodentium*, which was associated with an increased level of TωMCA and improved metabolic outcomes^47^. The proportion of circulating ωMCA was also increased in mice administered a polyphenol-rich camu-camu extract^69^. These mice were protected from diet-induced obesity, and it was suggested that their BA profile may be relevant to BAT activation and increased thermogenesis^69^. The Takeda G-protein receptor 5 (TGR5) is involved in several metabolic processes, and BA-TGR5 signaling is associated with BAT activation and energy expenditure^70^. Secondary BAs are the most potent TGR5 agonists, and their conjugation further increases their potency^71^. Hence, the BA profile of mice administered RebD may have increased the energy expenditure, which could explain why these mice gained less weight despite no changes in energy intake and fecal energy excretion.

BAs released in the digestive tract act as ligands for intestinal FXR, which plays a pivotal role in the negative feedback loop controlling BA synthesis by inhibiting the toxic accumulation of BA in the liver. Based on the mRNA expression, our data suggest that intestinal FXR signaling was enhanced in mice administered RebD and RebA. Although it has never been validated in vivo, a study using inverse virtual screening suggested that steviol could be an agonist of FXR^72^. In addition to its role in maintaining BA homeostasis, FXR also regulates the expression of several genes involved in metabolism. In the liver, FXR stimulation regulates lipid metabolism via inhibition of SREBP1c, which in turn decreases lipogenesis^66^. This is in line with our findings of decreased *acc* mRNA expression and reduced phosphorylation of Acc protein, the rate-liming step in fatty acid synthesis, in the livers of mice administered RebD. In addition, these mice presented with decreased levels of triglycerides and cholesterol in their livers, which can induce liver injury at high levels^73^. We also observed that mRNA expression of genes related to fatty acid oxidation tended to be decreased in the livers of RebD mice, which could suggest an early response to the molecule and thus contribute to reduced lipid availability for oxidation.

Lipopolysaccharides (LPS), a bacterial component of Gram-negative bacteria, can also induce inflammation in the liver^74^. We found that mice receiving RebD had decreased levels of LBP, which could suggest a decreased translocation of pro-inflammatory bacterial metabolites. In line with this, the level of TBARS, a marker of lipid peroxidation caused by reactive oxygen species, was also decreased in the livers of mice administered RebD. Thus, RebD reduced hepatic steatosis and decreased markers of inflammation. Although we did not observe any changes in the hepatic TG and cholesterol levels in mice administered RebA, another study found that RebA improved hepatic steatosis in diet-induced obese mice^75^. This discrepancy could be explained by a different length of intervention and diet composition. Another study using a genetic mouse model of obesity and insulin resistance showed that administration of RebA attenuated hepatic steatosis^76^. Overall, our data indicate that RebA does not further deteriorate hepatic health induced by an obesogenic diet and that RebD prevented steatosis and lipid peroxidation.

In conclusion, we have reported that chronic administration of the sweetener RebD decreased body weight gain and adiposity, reduced hepatic triglyceride and cholesterol accumulation, prevented lipid peroxidation in the liver, and decreased levels of LBP, a marker of metabolic endotoxemia. RebD administration also increased the abundance of *Faecalibaculum rodentium* and altered the composition and expression of genes related to bile acids, which are key players in metabolic homeostasis. Daily administration of RebA and RebD did not worsen glucose and insulin tolerance associated with an obesogenic diet. This data suggests that RebA and RebD are safe non-nutritive sweetener alternatives and do not deteriorate metabolic health in a well-recognized animal model of diet-induced obesity. More importantly, our data demonstrate that despite a strong structural similarity between RebA and RebD, they do not induce the same metabolic phenotypes in this obesity model, thus suggesting that RebD not only provides an improved taste but can also provide better health benefits than RebA, making the former steviol glycoside a better alternative as a natural sweetener.

### Supplementary Information

Supplementary Information 1.

Supplementary Figures.

Supplementary Information 2.

## Data Availability

The datasets used and/or analyzed during the current study are available from the corresponding author upon reasonable request.
