# Supplementary material for: Rebaudioside D decreases adiposity and hepatic lipid accumulation in a mouse model of obesity
Source: Sci Rep. 2024 Feb 6;14:3077. doi: 10.1038/s41598-024-53587-y (PMC10847429; doi:10.1038/s41598-024-53587-y)
Supplement: Supplementary file 1 — Supplementary Information 1. [file 41598_2024_53587_MOESM1_ESM.docx]

Explanation for WB

Explanation for the absence of images with different exposure time for our membranes ACC, pACC, and actin:

The images were exposed to the Blots – chemi protocol using the software Image Lab 5.1 (Bio-Rad). This preset program analyzes the membranes to take images when the intensity of the bands is ideal. The High Band Detection Sensitivity — sets detection at a higher level (75) for images that are fainter. Intense Bands exposure is optimized for all bands. Membranes exposed for longer period of time can be classified as overexposed when a red artifact can be seen on the bands as represented in Supplementary Figure 3. Image Lab can detect when an image is overexposed. Membranes exposed for shorter period of time can present faint bands making it difficult to discern differences between two similar bands. The images in Supplementary Figure 2 are not overexposed nor too faint since there is no red artifact. The different intensity between the bands can be seen and quantify.
