# Supplementary material for: Rebaudioside D decreases adiposity and hepatic lipid accumulation in a mouse model of obesity
Source: Sci Rep. 2024 Feb 6;14:3077. doi: 10.1038/s41598-024-53587-y (PMC10847429; doi:10.1038/s41598-024-53587-y)
Supplement: Supplementary file 2 — Supplementary Figures. [file 41598_2024_53587_MOESM2_ESM.pdf]

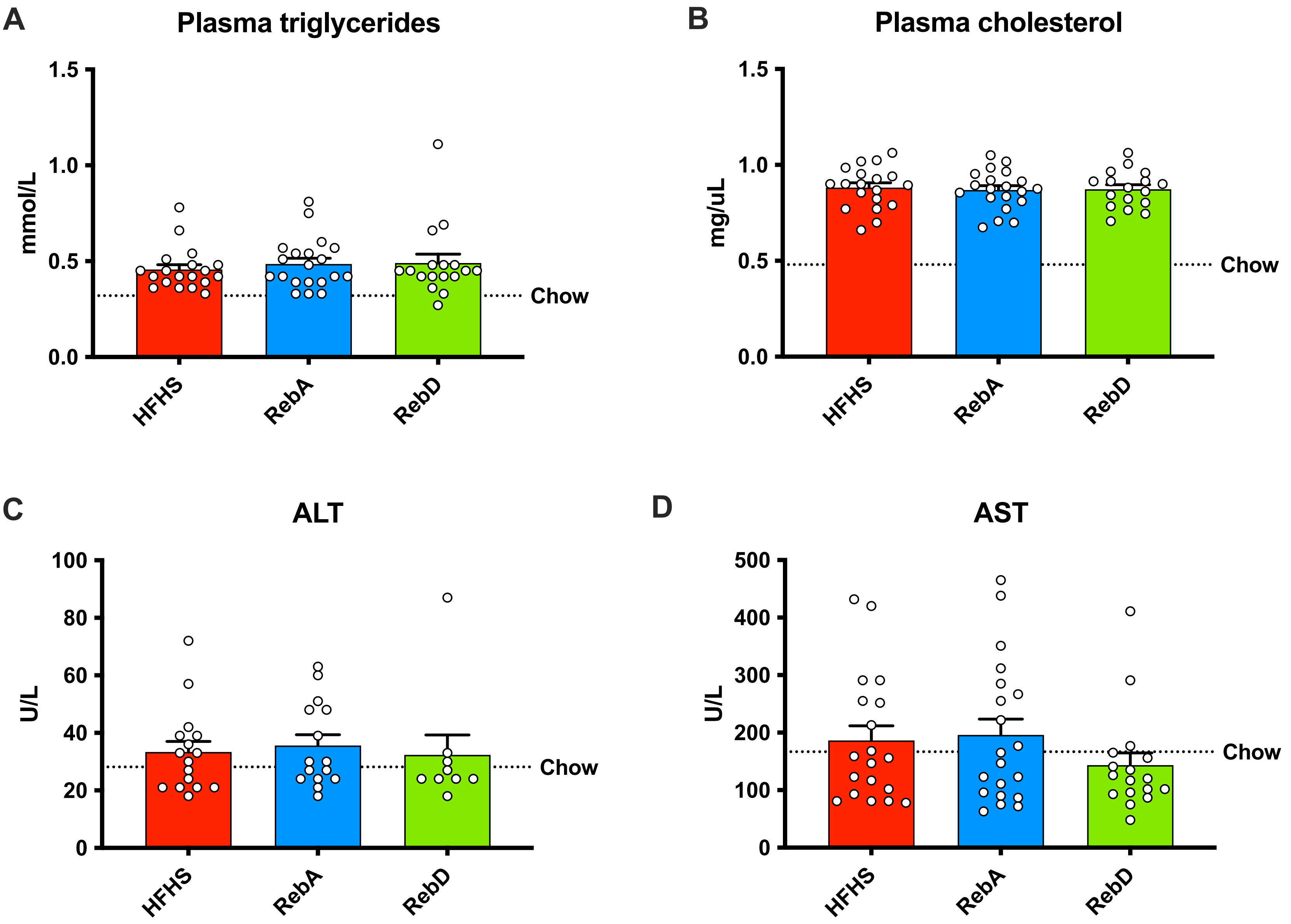

A
Plasma triglycerides
mmol/L
1.5
1.0
0.5
0.0
Chow
HFHS
RebA
RebD
B
Plasma cholesterol
mg/uL
1.5
1.0
0.5
0.0
Chow
HFHS
RebA
RebD
C
ALT
U/L
100
80
60
40
20
0
Chow
HFHS
RebA
RebD
D
AST
U/L
500
400
300
200
100
0
Chow
HFHS
RebA
RebD

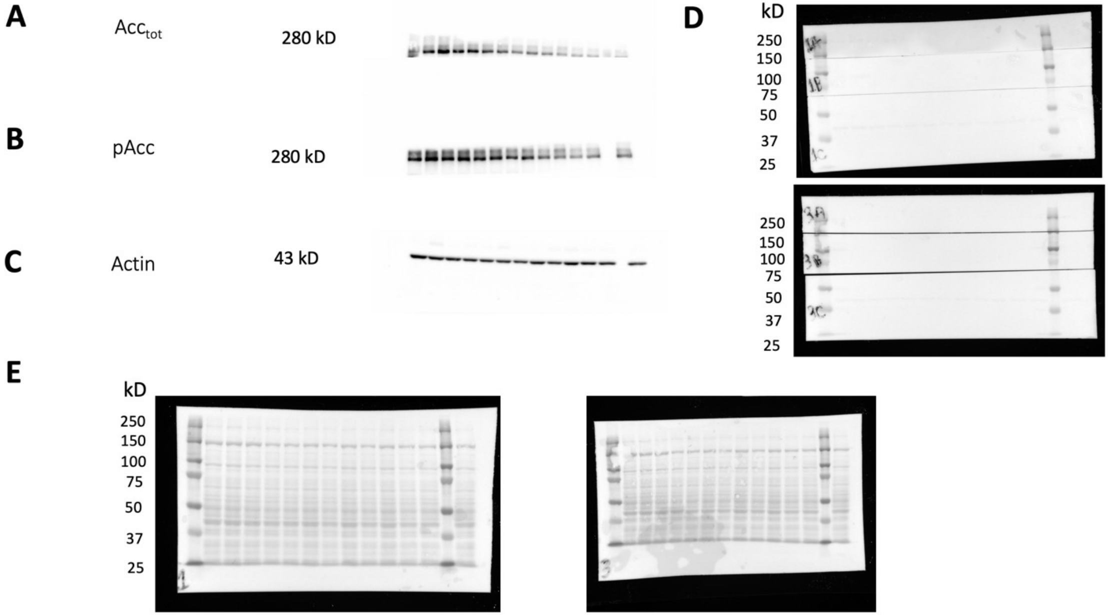
A
Acc$_{tot}$
280 kD
B
pAcc
280 kD
C
Actin
43 kD
D
kD
250
150
100
75
50
37
25
250
150
100
75
50
37
25
E
kD
250
150
100
75
50
37
25

A

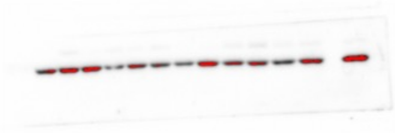

B

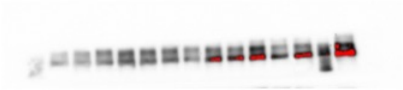

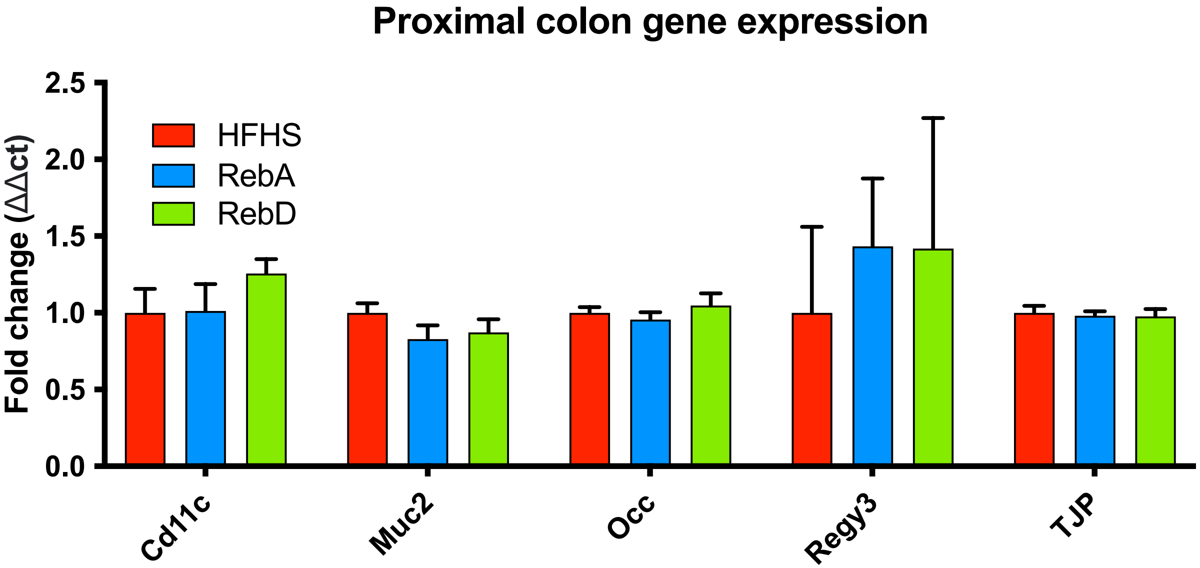
Proximal colon gene expression
Fold change (ΔΔct)
2.5
2.0
1.5
1.0
0.5
0.0
HFHS
RebA
RebD
Cd11c
Muc2
Occ
Regy3
TJP

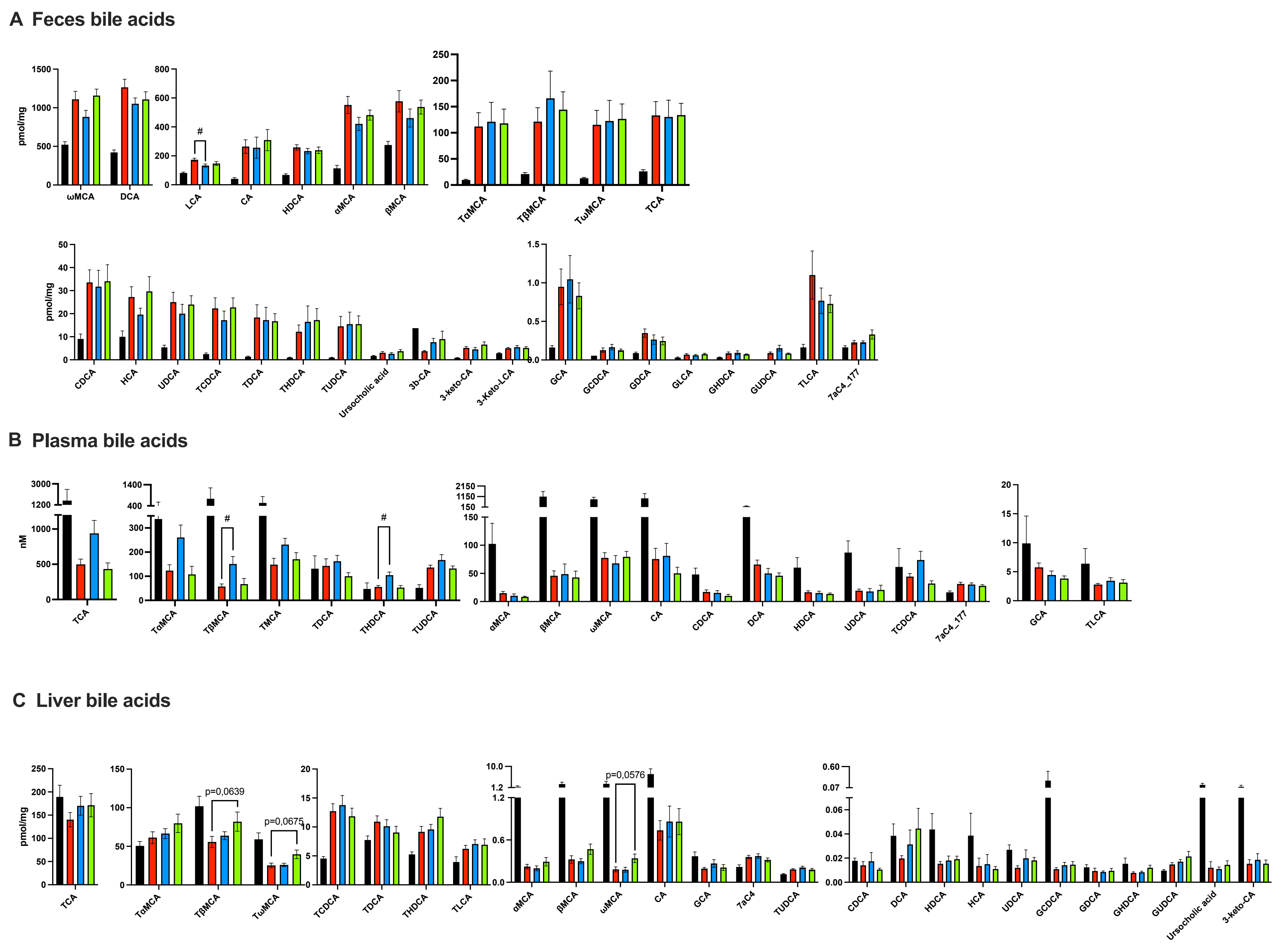
A Feces bile acids
pmol/mg
1500
1000
500
0
ωMCA
DCA
800
600
400
200
#
LCA
CA
HDCA
αMCA
βMCA
250
200
150
100
50
TαMCA
TβMCA
TωMCA
TCA
50
40
30
20
10
CDCA
HCA
UDCA
TCDCA
TDCA
THDCA
TUDCA
Ursocholic acid
3b-CA
3-keto-CA
3-Keto-LCA
1.5
1.0
0.5
0.0
GCA
GCDCA
GDCA
GLCA
GHDCA
GUDCA
TLCA
7aC4_177
B Plasma bile acids
nM
3000
1200
1000
500
TCA
1400
400
300
200
100
#
TαMCA
TβMCA
TMCA
TDCA
THDCA
TUDCA
2150
1150
150
100
50
αMCA
βMCA
ωMCA
CA
CDCA
DCA
HDCA
UDCA
TCDCA
7aC4_177
20
15
10
5
GCA
TLCA
C Liver bile acids
pmol/mg
250
200
150
100
50
TCA
150
100
50
p=0,0639
p=0,0675
TαMCA
TβMCA
TωMCA
20
15
10
5
TCDCA
TDCA
THDCA
TLCA
10.0
1.2
0.6
0.0
p=0,0576
αMCA
βMCA
ωMCA
CA
GCA
7aC4
TUDCA
0.60
0.07
0.06
0.04
0.02
0.00
CDCA
DCA
HDCA
HCA
UDCA
GCDCA
GDCA
GHDCA
GUDCA
Ursocholic acid
3-keto-CA

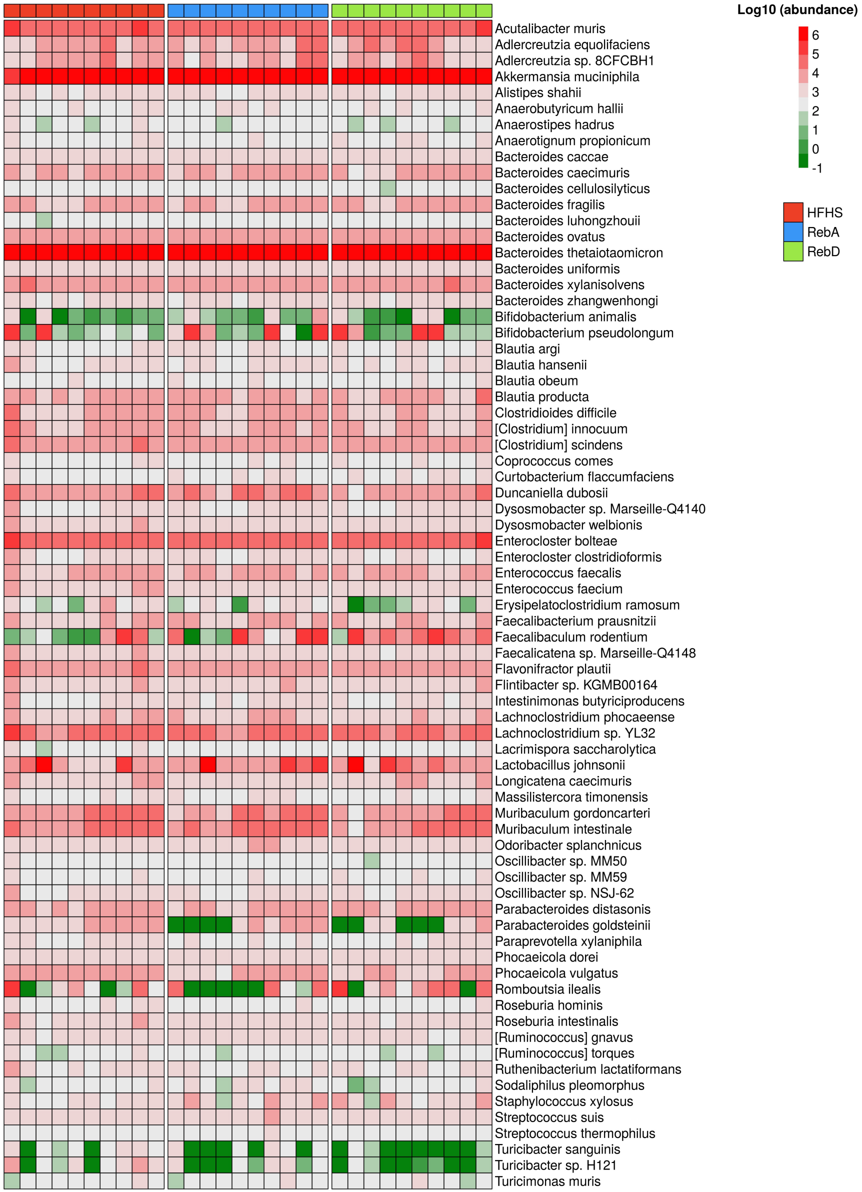
Log10 (abundance)
6
5
4
3
2
1
0
-1
HFHS
RebA
RebD
Acutalibacter muris
Adlercreutzia equolifaciens
Adlercreutzia sp. 8CFCBH1
Akkermansia muciniphila
Alistipes shahii
Anaerobutyricum hallii
Anaerostipes hadrus
Anaerotignum propionicum
Bacteroides caccae
Bacteroides caecimuris
Bacteroides cellulosilyticus
Bacteroides fragilis
Bacteroides luhongzhouii
Bacteroides ovatus
Bacteroides thetaiotaomicron
Bacteroides uniformis
Bacteroides xylanisolvens
Bacteroides zhangwenhongi
Bifidobacterium animalis
Bifidobacterium pseudolongum
Blautia argi
Blautia hansenii
Blautia obeum
Blautia producta
Clostridioides difficile
[Clostridium] innocuum
[Clostridium] scindens
Coprococcus comes
Curtobacterium flaccumfaciens
Duncaniella dubosii
Dysosmobacter sp. Marseille-Q4140
Dysosmobacter welbionis
Enterocloster bolteae
Enterocloster clostridioformis
Enterococcus faecalis
Enterococcus faecium
Erysipelatoclostridium ramosum
Faecalibacterium prausnitzii
Faecalibaculum rodentium
Faecalicatena sp. Marseille-Q4148
Flavonifractor plautii
Flintibacter sp. KGMB00164
Intestinimonas butyriciproducens
Lachnoclostridium phocaeense
Lachnoclostridium sp. YL32
Lacrimispora saccharolytica
Lactobacillus johnsonii
Longicatena caecimuris
Massilistercora timonensis
Muribaculum gordoncarteri
Muribaculum intestinale
Odoribacter splanchnicus
Oscillibacter sp. MM50
Oscillibacter sp. MM59
Oscillibacter sp. NSJ-62
Parabacteroides distasonis
Parabacteroides goldsteinii
Paraprevotella xylaniphila
Phocaeicola dorei
Phocaeicola vulgatus
Romboutsia ilealis
Roseburia hominis
Roseburia intestinalis
[Ruminococcus] gnavus
[Ruminococcus] torques
Ruthenibacterium lactatiformans
Sodaliphilus pleomorphus
Staphylococcus xylosus
Streptococcus suis
Streptococcus thermophilus
Turicibacter sanguinis
Turicibacter sp. H121
Turicimonas muris
