# Supplementary material for: Rebaudioside D decreases adiposity and hepatic lipid accumulation in a mouse model of obesity
Source: Sci Rep. 2024 Feb 6;14:3077. doi: 10.1038/s41598-024-53587-y (PMC10847429; doi:10.1038/s41598-024-53587-y)
Supplement: Supplementary file 3 — Supplementary Information 2. [file 41598_2024_53587_MOESM3_ESM.docx]

**Supplementary Table 1** List of primers used for Quantitative reverse transcriptase PCR (qPCR) analysis.

| **Gene** | **Forward** | **Reverse** |
| --- | --- | --- |
| Acc | GAAGTCAGAGCCACGGCACA | GGCAATCTCAGTTCAAGCCAGTC |
| Actin | CTCTAGACTTCGAGCAGGAG | AGAGTACTTGCGCTCAGGAG |
| Asbt | CAAACCTCAGAAGGACCAAACA | GTAGGAGGATTATTCCCGTTGTG |
| Bsep | AAGCTACATCTGCCTTAGACACAGAA | CAATACAGGTCCGACCCTCTCT |
| Cd11c | ACGTCAGTACAAGGAGATGTTGGA | ATCCTATTGCAGAATGCTTCTTTACC |
| cd11c | ACGTCAGTACAAGGAGATGTTGGA | ATCCTATTGCAGAATGCTTCTTTACC |
| Cpt1a | CAGCAAGATAGGCATAAACGC | AGTGTCCATCCTCTGAGTAGC |
| Cyp7a1 | AGCAACTAAACAACCTGCCAGTACTA | GTCCGGATATTCAAGGATGCA |
| Cyp7b1 | TGAGGTTCTGAGGCTGTGCTC | TCCTGCACTTCTCGGATGATG |
| F4/80 | TGCATCTAGCAATGGACAGC | GCCTTCTGGATCCATTTGAA |
| Fasn | CTCTGACAGGATTTGGGGTCAA | GCTGGCTCGTTCATGGGAT |
| Fgf15 | GAGGACCAAAACGAACGAAATT | ACGTCCTTGATGGCAATCG |
| Fgfr4 | GTACCCTCGGACCGCGGCACATAC | GCCGAAGCTGCTGCCGTTGATG |
| Fiaf | CAATGCCAAATTGCTCCAATT | TGGCCGTGGGCTCAGT |
| Fxr | CTGAGACTGGGTACCAGGG | CCATTCGCGGCTTCTTTGTC |
| Gadph | AATGGTGAAGGTCGGTGTG | GTGGAGTCATACTGGAACATGTAG |
| Hmgcr | GATCCGGAGGATCCAAGGACT | GTGGGAGGCCACGAAGAGG |
| Hprt | CCCCAAAATGGTTAAGGTTGC | AACAAAGTCTGGCCTGTATCC |
| Ibabp | GGTCTTCCAGGAGACGTGAT | ACATTCTTTGCCAATGGTGA |
| Muc2 | GGCCTCACCACCAAGCGTCC | TGGGCTGGCAGGTGGGTTCT |
| Ntcp | CAAACCTCAGAAGGACCAAACA | GTAGGAGGATTATTCCCGTTGTG |
| Occludin | TTTGGCTGCTCTTGGGTCTGTAT | ATGTCCGGCCGATGCTCTC |
| Osta | TGTTCCAGGTGCTTGTCATCC | CCACTGTTAGCCAAGATGGAGAA |
| Ostb | GATGCGGCTCCTTGGAATTA | GGAGGAACATGCTTGTCATGAC |
| Reg3γ | CCGTGCCTATGGCTCCTATTG | GCACAGACACAAGATGTCCTG |
| Shp | ACGATCCTCTTCAACCCAGA | AGGGCTCCAAGACTTCACAC |
| Srebp1c | AAGCTGTCGGGGTAGCGTCT | GCTGGAGCATGTCTTCAAATGTG |
| Tgr5 | CTGCTGGCTGCTTCTTCC | CACTGCCATGTAGCGTTCC |
| Tjp1 | GCCACTACAGTATGACCATCC | AATGAATAATATCAGCACCATGCC |
| Cyp27a1 | PT 58.42377566 |  |
| Cyp2c70 | PT 58.7509415 |  |
| Cyp8b1 | PT 58.12268653.g |  |

**Supplementary Figure 1** (A) Plasma triglyceride, (B) Plasma cholesterol, (C) ALT, and (D) AST measured in the livers of HFHS, RebA and RebD mice.

**Supplementary Figure 2** Original unprocessed images of the membranes used in Figure 2J for (**A**) Total ACC, (**B**) pACC, and (**C**) Actin. The membranes were cut before the hybridization with antibodies. The membranes are exposed to chemiluminescence where the band of interest can be seen. The membranes were exposed for (**A**) 1,683, (**B**) 6,815, and (**C**) 2,877 seconds using the blots – chemi protocol. Parameters were already preset with the software Image Lab 5.1 (Bio-Rad), no modifications were made in the protocols for imaging. (**D**) Reconstitution of the original membranes used in Figure 2J as they appeared before they were cut exposed to colorimetric. The middle part of both membranes and the bottom part of the membrane on the right were not used for the Figure 2J. The middle part of both membranes was used for other proteins that were not detected under the tested conditions. The bottom part of the membrane on the right was used for the quantification of actin but was not shown in the Figure 2J. (**E**) Membranes with ponceau S as the membrane appeared before it was cut in colorimetric.

**Supplementary Figure 3.** Two examples of overexposed membranes in chemiluminescence obtained with the software Image Lab 5.1. Membranes were both exposed for 250 seconds manually without using the preset program blots – chemi protocol.

**Supplementary Figure 4.** Proximal colon mRNA expression of genes related to intestinal permeability. Data are expressed as the mean ± SEM. n=17-20.

**Supplementary Figure 5.** Bile acid profile in (A) feces, (B) plasma, and (C) liver. αMCA, α-murocholic acid; βMCA, β-murocholic acid; CA, cholic acid; CDCA, chenodeoxycholic acid; DCA, deoxycholic acid; GCA, glycocholic acid; GCDCA, glycochenodeoxycholic acid; GLCA, glycolithocholic acid;GHDCA, glycohyodeoxycholic acid; GUDCA, glycoursodeoxycholic acid;HCA, hyocholic acid; HDCA, Hyodeoxycholic acidωMCA, ω-murocholic acid; UDCA, ursodeoxycholic acid; LCA, lithocholic acid; TLCA, tauro-lithocholic acid; TCDCA, taurochenodeoxycholic acid; TDCA, taurodeoxycholic acid; TUDCA, tauro-ursodeoxycholic acid; THDCA, tauro-hyodeoxycholic acid; UDCA, ursodeoxycholic acid;TαMCA, tauro-α-murocholic acid; TβMCA, tauro-β-murocholic acid; TωMCA, tauro-ω-murocholic acid; TCA, tauro-cholic acid.

**Supplementary Figure 6.** Heatmap of the bacterial species abundance after the dietary intervention (the color scale represents the log_10_ transformed abundance)
